# Arrhythmia classification based on multi-input convolutional neural network with attention mechanism

**DOI:** 10.1371/journal.pone.0326079

**Published:** 2025-06-17

**Authors:** Bin Zheng, Wenbo Luo, Mingming Zhang, Huiyuan Jin

**Affiliations:** 1 School of Mathematics, Statistics and Mechanics, Beijing University of Technology, Beijing, China; 2 Zhengzhou Aerotropolis Institute of Artificial Intelligence, Zhengzhou, China; Universiti Tunku Abdul Rahman, MALAYSIA

## Abstract

Arrhythmia is a prevalent cardiac disorder that can lead to severe complications such as stroke and cardiac arrest. While deep learning has advanced automated ECG analysis, challenges remain in accurately classifying arrhythmias due to signal variability, data imbalance, and feature representation limitations. In this work, we propose a novel arrhythmia classification algorithm based on a multi-input convolutional neural network (CNN) enhanced with a Squeeze-and-Excitation (SE) attention mechanism. Distinct from previous methods that rely on single-resolution features or unimodal inputs, our model integrates multi-scale time-frequency representations derived from Short-Time Fourier Transform (STFT) applied to ECG signals segmented into two temporal resolutions. The dual-branch CNN architecture enables complementary feature learning from both short and long segments, while SE blocks enhance inter-channel dependencies to prioritize critical features. The fusion strategy combines feature maps via bicubic interpolation and element-wise summation to maintain spatial integrity. Evaluated on MIT-BIH and SPH arrhythmia databases, the proposed model achieves high accuracy (99.13% and 95.84%, respectively) and Macro-F1 scores (94.46% and 95.91%), outperforming several state-of-the-art approaches. These results highlight the model’s potential for robust and interpretable arrhythmia classification in clinical practice.

## 1. Introduction

Cardiovascular disease, a leading cause of mortality, poses a significant public health challenge [1]. One of the key tools in diagnosing cardiovascular conditions is the electrocardiogram (ECG), which records the electrical activity of the heart. By analyzing ECG patterns, healthcare professionals can detect arrhythmias, which are abnormalities in the heart’s rhythm [2–3]. These detections are crucial for early intervention and treatment, potentially reducing the risk of severe cardiovascular events.

At present, deep learning has been widely applied in ECG signal classification, leveraging neural networks to automatically extract and learn the characteristics of ECG signals. When performing arrhythmia classification using Convolutional Neural Networks (CNNs), researchers employ various strategies to process ECG signals. One approach is to use raw ECG signals without any noise reduction [4–6]. Another approach involves applying wavelet transform to reduce noise [7–8], resulting in one-dimensional ECG signal sequences suitable for input into one-dimensional CNN models. Additionally, ECG signals can be converted into two-dimensional images, such as time-domain maps or time-frequency spectrum diagrams, and analyzed using two-dimensional CNN models. By using the Short-Time Fourier Transform (STFT), the ECG signal is transformed into a time-frequency spectrum diagram, which can be utilized by two-dimensional CNNs [9–10]. Converting ECG signals into two-dimensional grayscale or RGB images has been shown to achieve higher classification accuracy [11–12]. Some researchers generate n-BSM images by transforming scalograms from continuous wavelet transform into beat score vectors for arrhythmia classification [13]. Furthermore, some researchers opt to convert ECG signals into different forms, such as grayscale images and scalograms, and analyze them using bimodal CNNs, which combine and process multiple forms of input to enhance classification performance [14–15]. These diverse approaches highlight the flexibility and effectiveness of deep learning techniques in improving the accuracy of arrhythmia detection from ECG signals.

The integration of attention mechanisms with deep neural networks has become a trend in ECG signal classification [16–24]. The SE-ECGNet model described in [16] combines the residual network and the squeeze-and-excitation (SE) module, resulting in superior performance compared to state-of-the-art models. A deep neural network model was built employing the ResNet architecture along with the SE module for the classification of 12-lead ECG data [17]. The attention mechanism was incorporated into a dual-channel deep neural network to merge important features from the 12 leads, achieving accurate classification of nine types of arrhythmia signals [18]. Moreover, some researchers incorporate various channel attention mechanisms to further enhance model performance [19–24]. These advancements illustrate how attention mechanisms can significantly improve the accuracy and efficiency of ECG signal classification by enabling models to prioritize relevant features and handle complex data relationships.

In recent years, fused neural network models that combine CNN with Recurrent Neural Networks (RNN) have been proposed for ECG classification [25–33]. The fusion of RNN and CNN can address the CNN model’s limitation in capturing the temporal autocorrelation of ECG signal sequences. A multi-input arrhythmia classification model was developed to integrate both CNN and Bidirectional Long Short-Term Memory (BiLSTM) networks [25]. The model takes ECG signals segmented into two time-window lengths: 0.75 seconds and 4 seconds. By doing so, it utilizes the strengths of each network: CNNs for local, small-scale features and BiLSTM networks for extracting large-scale features over longer time periods. This fusion provides benefits compared to using a standalone network model. While result in [26] favors one-dimensional ECG signal-based networks over two-dimensional representations and fusion models, the potential of fusion models in certain cases is also acknowledged. Moreover, the attention mechanism can be integrated into these fused neural network models to further enhance their performance [29]. Furthermore, [30] introduced a novel method to more effectively extract heartbeat differences by constructing time representation input, which is then processed by the CNN-LSTM-Attention model.

Recent research has made significant strides in applying deep learning techniques to ECG signal analysis. However, a critical gap remains in understanding how multi-scale temporal features can be effectively fused with attention mechanisms to improve arrhythmia classification accuracy. Existing works such as SE-ECGNet [16] and others employing channel attention [17–23] have demonstrated promising results, but often rely on single-scale inputs or fixed-length ECG segments, potentially limiting the model’s ability to generalize across different types of arrhythmias.

Moreover, some recent studies, such as the arrhythmia classification method based on a multi-head self-attention mechanism proposed in [22], focus heavily on attention architectures but do not leverage dual-scale or multi-input strategies. Our method builds upon and extends these efforts by incorporating both short and long time-window ECG segments using STFT, which are then processed through a parallel multi-input CNN architecture with integrated SE blocks. This design enables enhanced extraction of local and contextual features relevant to arrhythmia classification. To the best of our knowledge, our proposed SE-multi-input CNN model is the first to combine dual-scale STFT-based ECG representations with attention-enhanced CNNs in a unified architecture. This hybrid approach aims to address current limitations in capturing multi-scale dynamics of ECG signals and demonstrates superior performance across benchmark datasets.

The rest of this paper is structured as follows: Section 2 describes the ECG dataset used and data preprocessing of the ECG signals. In Section 3, the proposed SE-multi-input CNN model is introduced. In Section 4, we present the evaluation metrics and discuss model performance. In Section 5, the conclusion is given.

## 2. Dataset used and preprocessing

### 2.1. Database

The study utilizes two key arrhythmia databases: the MIT-BIH arrhythmia database [34], sourced from the Massachusetts Institute of Technology, renowned globally for its standard ECG datasets. Comprising 48 dual-lead ECG recordings spanning from 1975 to 1979, each lasting slightly over 30 minutes with a unified sampling frequency of 360 Hz, it features data from 47 subjects. These subjects, aged 23 to 89, are divided into two groups: 23 representative samples for routine clinical testing and 25 complex samples for challenging arrhythmia testing. Heartbeats in the MIT-BIH database are classified into five types according to ANSI/AAMI EC57–2012 standards: N, S, V, F, and Q.

The second database, the Shaoxing People’s Hospital arrhythmia database (SPH) [35], is a collaboration between Chapman University and Shaoxing People’s Hospital, housing 10,646 12-lead ECGs from unique patients. The recordings, with a sampling rate of 500 Hz and each lasting 10 seconds, encompass 11 common rhythms and 67 additional cardiovascular conditions. This database includes both original and noise-reduced ECG data, with 41 recordings excluded due to incompleteness or zero content. In [35], there are 11 rhythms categorized into four heartbeat types: AFIB, GSVT, SB, and SR.

### 2.2. Preprocessing of MIT-BIH arrhythmia database

#### 2.2.1 Signal selection and noise removal.

The MIT-BIH arrhythmia database comprises 48 dual-lead datasets, from which we retain 45 modified limb lead II (MLII) recordings, excluding data 102, 104, and 114.

During ECG signal collection, susceptibility to various noise interferences, including industrial frequency, baseline drift, and electromyographic (EMG) interference, is evident. Employing a wavelet threshold noise reduction algorithm [36–37], we filter out industrial frequency and EMG interferences through an eight-layer wavelet decomposition using Daubechies 4 (DB4), adopting Stein’s non-alternate estimation for threshold selection. Additionally, we employ a median filter with a neighborhood size of 108 (30% of the sampling frequency) [38–39] to address baseline drift.

**Fig 1:**
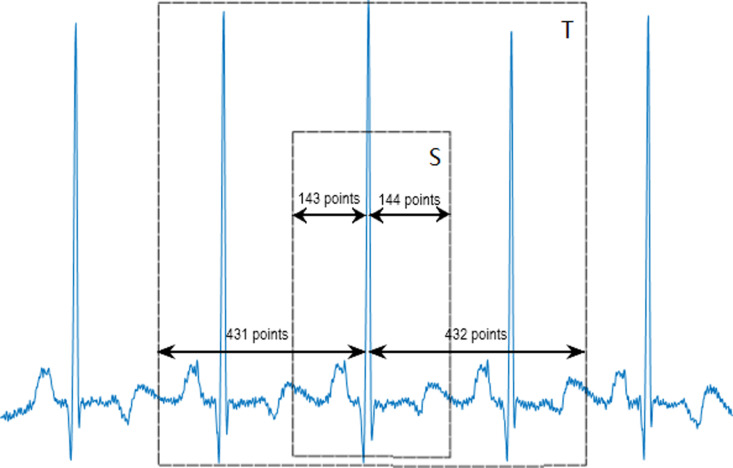
Segmentation of single-cycle and three-cycle length heartbeats.

Within the MIT-BIH arrhythmia database, each cardiac cycle comprises 288 sampling points. Signal cropping involves selecting 143 sampling points to the left and 144 sampling points to the right of each R-peak for single-cycle length (denoted by S) and 431 sampling points to the left and 432 sampling points to the right for three-cycle length (denoted by T), as depicted in Fig 1.

Due to the non-stationarity of ECG signals, characterized by time-varying statistical attributes, such as mean and variance, we utilize Short-Time Fourier Transform (STFT) for time-frequency representation. Utilizing the Kaiser window function with a size of 0.2s (72 sampling points) and 0.01s increment, we compute the time-frequency spectrogram of ECG signals by:


$$X_{STFT}(t,\omega )=\sum_{n=0}^{L-1}x(n)w(n-t)e^{-j\omega n},$$

where *L* is the window length, x(n) is the input signal, w(n) is the Kaiser window function defined by


$$\begin{array}{c} w(n)=\left\{ \begin{array}{c} \frac{I_{0}(\pi \alpha \sqrt{1-{\left(\frac{2n}{N-1}-1\right)}^{2}})}{I_{0}(\pi \alpha )},\;0\leq n\leq N-1\\ 0\;\;,\;otherwise.\; \end{array}\right.\; \end{array}$$

**Fig 2:**
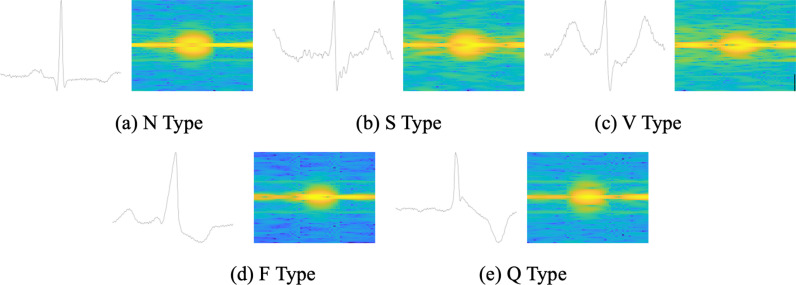
Time-domain (original) and time-frequency diagrams (after STFT) of single-cycle heartbeats.

After applying STFT, each single-cycle and three-cycle ECG signal transforms into a spectrogram image sized 72 × 55 and 72 × 199, respectively, as illustrated in Fig 2.

Each single-cycle ECG signal is paired with a three-cycle signal, aligning the former at the center of the latter, and labeled accordingly. Following data preprocessing, we obtain 82,005 N beats, 2,762 S beats, 7,177 V beats, 798 F beats, and 3,885 Q beats, totaling 96,627 beats.

We split the dataset into 90% training and 10% testing, following [40]. This higher training proportion aids in countering class imbalance and stabilizing training.

#### 2.2.2. Data imbalance and augmentation.

The MIT-BIH database displays a notable variance in the occurrence of different heartbeat types, leading to a significant data imbalance. Approximately 84.87% of samples are of the N type, while S, V, F, and Q types represent 2.86%, 7.43%, 0.83%, and 4.02% respectively. This imbalance can lead to inaccuracies in feature extraction, potentially resulting in lower classification rates for specific heartbeat categories.

Table 1 illustrates the sample distribution in both the original training and test sets. To mitigate this issue, we employ an effective data augmentation technique known as Mixup, introduced by Zhang et al. in 2017 [41]. Mixup, a form of Vicinal Risk Minimization (VRM), generates new training samples and labels through linear interpolation [42, 43]. Mixup involves sampling a mixing coefficient from a Beta distribution, utilizing original input vectors and their corresponding one-hot label encodings. The formula for Mixup is:

**Table 1:** The number of samples in the original training set and the test set.

Heartbeat type	Number of samples	
	Training set (90%)	Test set (10%)
N	79519	8835
S	2486	276
V	6459	718
F	718	80
Q	3496	389


$$\begin{array}{cc} & \tilde{x}=\varepsilon x_{a}+(1-\varepsilon )x_{b},\\ & \tilde{y}=\varepsilon y_{a}+(1-\varepsilon )y_{b}, \end{array}$$

where mixing coefficient ε is sampled from β(1,1) distribution, $x_{a}$, $x_{b}$ are the original input vectors, $y_{a}$, $y_{b}$ are the one-hot label encodings.

Each sample in our combined dataset comprises two ECG signals of different lengths. Mixup is applied specifically to three-cycle ECG signals, with their middle cycles extracted as paired single-cycle signals. Augmentation focuses on S, V, and F types, with generation ratios of 1, 0.5, and 2 respectively, as depicted in Table 2, showcasing the sample distribution in the training set before and after augmentation.

**Table 2:** Number of samples in training set for each heartbeat type before and after data augmentation.

Heartbeat type	Number of samples in training set	
	Before data augmentation	After data augmentation
N	79519	79519
S	2486	4972
V	6459	9689
F	718	2154
Q	3496	3496

### 2.3. Preprocessing of SPH arrhythmia database

The SPH arrhythmia database contains noise-reduced data but lacks R-peak annotations [35]. Hence, we employ the R-peak detection algorithm from [44] to precisely identify R-peak positions in the signal. Subsequently, we implement adaptive cropping to accommodate the diverse heart rates observed across different patients. The number of sampling points per cardiac cycle $N_{s}$ for each patient is calculated by


$$N_{s}=\frac{60}{H_{r}}\times S_{r},$$

where $H_{r}$ denotes heart rate, and $S_{r}$ is sampling rate.

**Fig 3:**
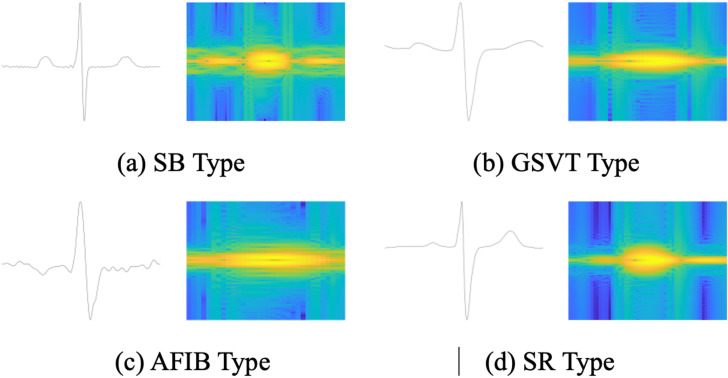
Time-domain (original) and time-frequency diagrams (after STFT) of single-cycle heartbeats.

For every R-peak detected, we extract a single-cycle ECG signal by sampling *round*($N_{s}$/2) points on each side, and a three-cycle ECG signal by sampling round(3×$N_{s}$/2) points on each side. These signals are then resized using bi-cubic interpolation to 1 × 400 and 1 × 1200, respectively. Compared to conventional interpolation methods, Bi-cubic interpolation better preserves image details and texture information during processing. Utilizing STFT, we represent the signals in the time-frequency domain, recommending the use of a Kaiser window function with a size of 0.2s and 0.01s increment. Following STFT, each single-cycle and three-cycle ECG signal transforms into a spectrogram image sized 100 × 61 and 100 × 221, respectively. Fig 3 illustrates the time-domain plot and time-frequency spectrum diagram of the single-cycle signal for each heartbeat type.

Following a methodology similar to the MIT-BIH fusion dataset, single-cycle ECG signals are paired with three-cycle signals, and organized into an 80% training and 20% testing split. This ratio aligns with standard practice in SPH-based studies and ensures sufficient samples per class. This preprocessing yields the SPH fusion dataset, with balanced representation of heartbeat types (Table 3), obviating the need for data augmentation.

**Table 3:** The numbers of different types in SPH fusion dataset.

Merged name	Total	Training data size (80%)	Testing data size (20%)
SB	23921	19137	4784
GSVT	40540	32432	8108
AFIB	29473	23578	5895
SR	21198	16958	4240
All	115132	92105	23027

## 3. Methodology

### 3.1. Method overview

This paper presents a novel deep learning framework for arrhythmia classification based on multi-scale two dimensional ECG spectrogram analysis. The proposed model—termed SE-multi-input CNN—leverages dual time-frequency representations generated by Short-Time Fourier Transform (STFT), processed via a two-branch CNN architecture with Squeeze-and-Excitation (SE) blocks embedded in each branch. The branches are designed to capture fine-grained and contextual ECG features from short and long cardiac segments, respectively. A key novelty of our approach lies in the multi-scale feature fusion mechanism using bicubic interpolation and element-wise summation, which preserves spatial consistency across branches without introducing redundancy.

### 3.2. Model architecture

**Fig 4:**
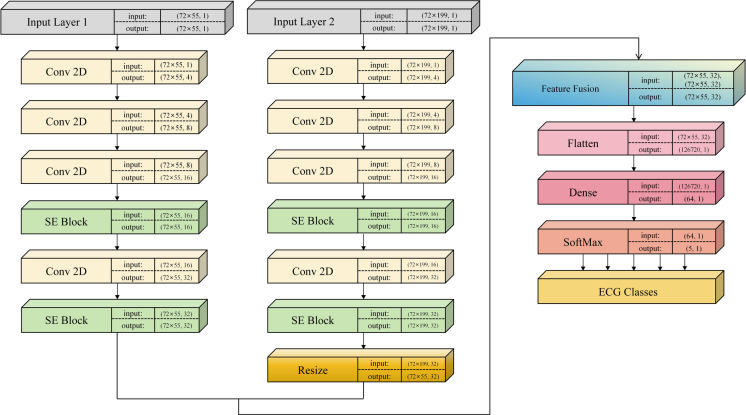
The architecture of the SE-multi-input CNN model.

This section focuses on crafting a multi-input CNN model featuring Squeeze-and-Excitation (SE) blocks for arrhythmia classification, as illustrated in Fig 4. The model employs two parallel CNN branches with convolution kernels of varying sizes to capture multi-scale features from single-cycle and three-cycle signals. This approach enables the extraction of both local features from the current heartbeat and correlation features from adjacent heartbeats. Additionally, two SE blocks are integrated into each branch to dynamically modulate the significance of features across different channels, thus enhancing the classification accuracy of the model.

#### 3.2.1. Multi-input CNN.

CNN architecture has demonstrated remarkable efficacy in ECG signal classification, leveraging multiple nonlinear filters to extract diverse local features by correlating adjacent pixels. To enhance classification accuracy, we devise a multi-input CNN model with an attention mechanism, facilitating the capture of multi-scale features from both single-cycle and three-cycle ECG signals.

The convolutional layers of the parallel CNN branches utilize distinct kernel sizes to capture a range of temporal local dependencies. The small-scale branch processes single-cycle signals, employing four convolutional layers with filter sizes of 3 × 3 and filter counts of 4, 8, 16, and 32 respectively, followed by Rectified Linear Unit (ReLU) activation. Similarly, the large-scale branch operates on three-cycle signals, employing four convolutional layers with filter counts mirroring those of the small-scale branch and a filter size of 5 × 5.

SE blocks are introduced in each CNN branch to recalibrate the generated feature maps, enhancing feature extraction performance (see section 3.2.2 for details). The features from both branches are fused via element-wise summation, necessitating adjustment of the three-cycle signal’s feature map size via bicubic interpolation. Subsequently, a flatten layer converts the fused features into one-dimensional data for the subsequent fully connected layers. The first fully connected layer comprises 64 neurons, while the second corresponds to the total number of categories to be classified, i.e., 5 in the MIT-BIH arrhythmia database and 4 in the SPH arrhythmia database, utilizing softmax activation.

In this study, the model is trained for 30 epochs with a batch size of 256, using the Adam optimizer with a learning rate of 0.001. Feature normalization is applied both before input and after fusion to accelerate model convergence and enhance generalization.

#### 3.2.2. Squeeze-and-Excitation block.

To implement channel attention, we integrate two SE blocks within each CNN branch. This incorporation enables the selective enhancement of crucial features conducive to arrhythmia classification while suppressing less relevant features, thereby enhancing model accuracy.

**Fig 5:**
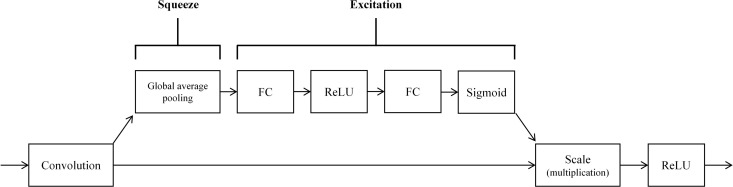
The structure of SE block used in the SE-multi-input CNN model.

The SE block structure employed in the SE-multi-input CNN model is depicted in Fig 5. Comprising two steps, the SE block begins with the squeeze operation, which compresses each feature channel into a single value via global average pooling of the feature map. Subsequently, the excitation operation utilizes these values as initial weights, generating new weights through two fully connected layers and activation functions. Finally, these new weight values are multiplied with the original features.

Within each CNN branch, the two SE blocks collectively contain four fully connected layers, with the number of neurons specified as 8, 16, 16, and 32 respectively.

## 4. Results

### 4.1. Evaluation metrics

This paper employs five evaluation metrics to assess model performance: Accuracy (Acc), Sensitivity (Sen), Positive Predictive Value (PPV), F1-score, and Macro-F1 score. The calculation formulas are provided below:


$$\begin{array}{c} Acc=\frac{TP+TN}{TN+FP+TP+FN}\times 100\%,\\ \;\;Sen=\frac{TP}{TP+FN}\times 100\%,\\ PPV=\frac{TP}{TP+FP}\times 100\%,\\ F1=\frac{2PPV\times Sen}{PPV+Sen}\times 100\%,\\ Macro-F1=\frac{\sum_{i=1}^{L}{F1}_{i}}{L}\times 100\%, \end{array}$$

where TP indicates True Positive, TN indicates True Negative, FP indicates False Positive, FN indicates False Negative, the value of L is 5 in the MIT-BIH arrhythmia database and is 4 in the SPH arrhythmia database, ${F1}_{i}$ denotes the F1-score of the *i* th classification. After computing sensitivity (or PPV) for each category, the average sensitivity (or average PPV) can be obtained by taking the arithmetic mean, particularly beneficial for assessing multi-class classification on imbalanced datasets.

### 4.2. Classification results for the MIT-BIH arrhythmia database

#### 4.2.1. Comparison of results before and after data augmentation.

**Fig 6:**
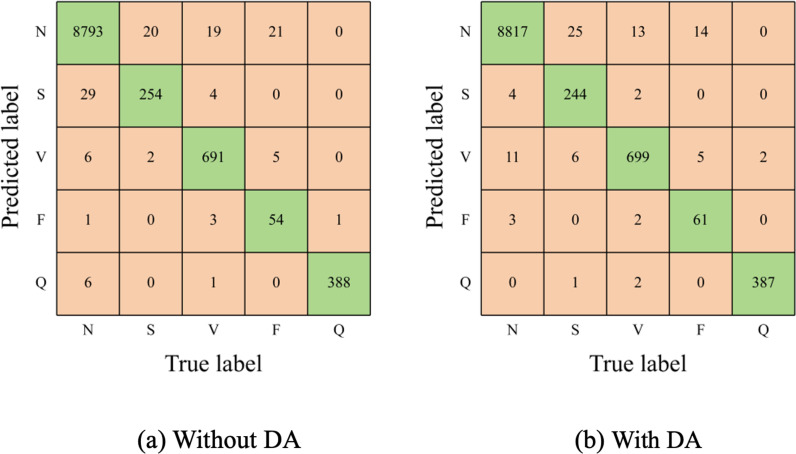
Confusion matrices for SE-multi-input CNN with/without data augmentation (DA).

Fig 6 displays the confusion matrices of the proposed SE-multi-input CNN model, with and without data augmentation (DA). Table 4 summarizes the accuracy, average sensitivity, average PPV, and Macro-F1 score. The results indicate the model’s outstanding performance in terms of overall accuracy across all types and the accuracy of individual types. Incorporating DA generally improves most metrics, except for sensitivity in the S type and PPV in the V type. Specifically, the relatively lower sensitivity in the S type results from misclassifications of some S beats as either N or V beats. Similarly, the lower PPV in the V type stems from misclassifications of some N and S beats as V beats.

**Table 4:** Comparison of SE-multi-input CNN without (and with) data augmentation.

Heartbeat Type	Acc	Sen	PPV	F1
N	99.01% (99.32%)	99.52% (99.80%)	99.32% (99.41%)	99.42% (99.60%)
S	99.47% (99.63%)	92.03% (88.41%)	88.50% (97.60%)	90.23% (92.78%)
V	99.61% (99.58%)	96.24% (97.35%)	98.15% (96.68%)	97.19% (97.02%)
F	99.70% (99.80%)	67.50% (76.25%)	91.53% (92.42%)	77.70% (83.56%)
Q	99.92% (99.95%)	99.74% (99.49%)	98.23% (99.23%)	98.98% (99.36%)
All	98.85% (99.13%)	91.01% (92.26%)	95.15% (97.07%)	92.70% (94.46%)

#### 4.2.2. Comparison between multi-input model and single-input model.

**Fig 7:**
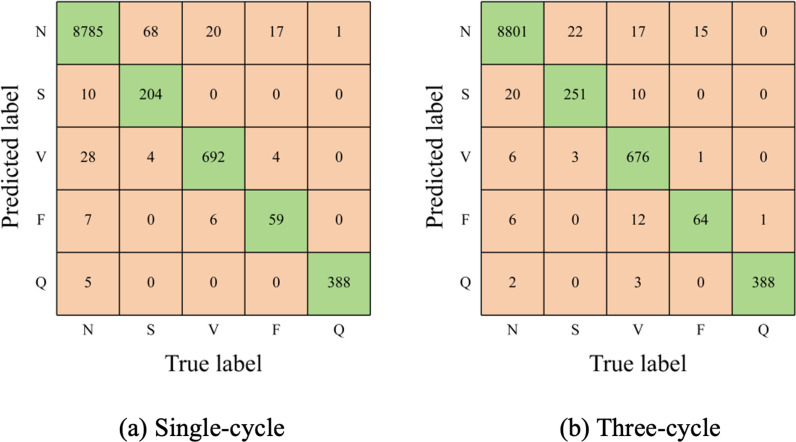
Confusion matrices for single-input CNN models.

In this section, we assess the performance of the SE-multi-input CNN alongside two other CNN models. One CNN model utilizes the smaller-scale branch of the SE-multi-input CNN to analyze single-cycle signals, while the other corresponds to the larger-scale branch designed for three-cycle signals. Results from Table 5 demonstrate that the SE-multi-input CNN model outperforms others in terms of overall accuracy, average PPV, and Macro-F1 score. However, its average sensitivity slightly lags behind the CNN tailored for three-cycle signals. Further analysis, comparing confusion matrices in Fig 7 and Fig 6(b), reveals this sensitivity discrepancy stems from an increase in S beats misclassified as N or V beats.

**Table 5:** Comparison of multi-input CNN and single-input CNN.

Models	Overall Acc	Average Sen	Average PPV	Macro-F1
CNN for single-cycle	98.35%	88.64%	93.97%	90.99%
CNN for three-cycle	98.85%	92.89%	92.62%	92.74%
SE-multi-input CNN	99.13%	92.26%	97.07%	94.46%

#### 4.2.3. Comparison between SE-multi-input CNN model and Multi-input CNN model.

**Fig 8:**
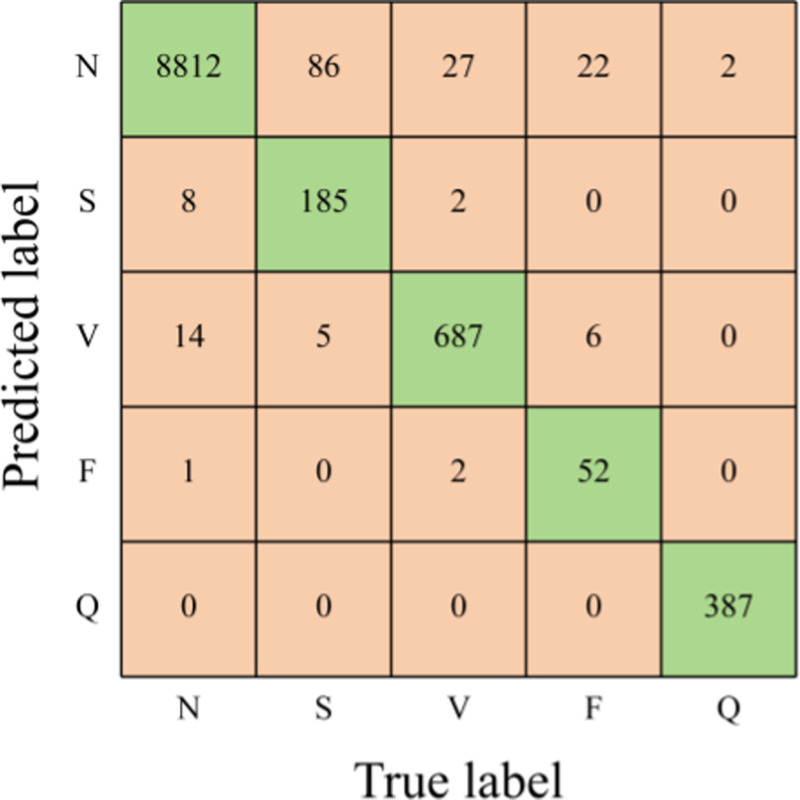
Confusion matrix for the Multi-input CNN model.

Table 6 highlights the superiority of the SE-multi-input CNN model over the Multi-input CNN model across all evaluation metrics. Notably, the overall accuracy, average sensitivity, average PPV, and Macro-F1 score exhibit improvements of 0.83%, 6.87%, 0.19%, and 4.36%, respectively. A comparison between the confusion matrices in Fig 8 and Fig 6(b) illustrates that incorporating the attention mechanism enhances the accuracy of classifying ECG signals, particularly for S, V, and F types.

**Table 6:** Comparison of SE-multi-input CNN with Multi-input CNN.

Models	Overall Acc	Average Sen	Average PPV	Macro-F1
Multi-input CNN	98.30%	85.39%	96.88%	90.10%
SE-multi-input CNN	99.13%	92.26%	97.07%	94.46%

### 4.3. Classification results for the SPH arrhythmia database

#### 4.3.1. Comparison between multi-input model and single-input model.

**Fig 9:**
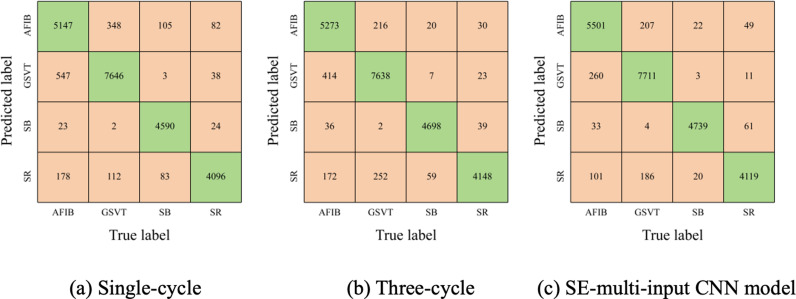
Confusion matrices for two single-input model and SE-multi-input CNN models.

Below, we assess the performance of the SE-multi-input CNN model using the SPH database. As depicted in Table 7, it outperforms two single-input CNN models across all evaluation metrics. Furthermore, examination of the confusion matrices in Fig 9 reveals notable enhancements in accuracy for identifying AFIB, GSVT, and SB types. Interestingly, the SE-multi-input CNN model exhibits superior performance on the SPH database compared to the MIT-BIH database, possibly due to the less severe data imbalance issue in the SPH database.

**Table 7:** Comparison of multi-input CNN and single-input CNN.

Models	Overall Acc	Average Sen	Average PPV	Macro-F1
CNN for single-cycle	93.28%	93.54%	93.50%	93.49%
CNN for three-cycle	94.48%	94.92%	94.42%	94.60%
SE-multi-input CNN	95.84%	96.16%	95.70%	95.91%

#### 4.3.2. Comparison between SE-multi-input CNN model and Multi-input CNN model.

**Fig 10:**
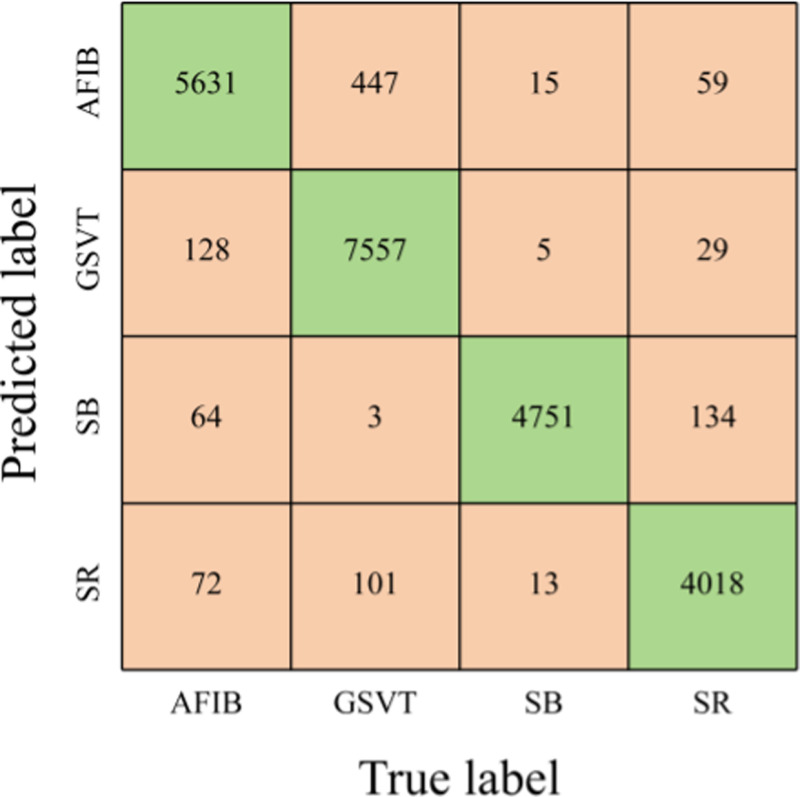
Confusion matrix for the Multi-input CNN model.

Just like the findings with the MIT-BIH dataset, incorporating an attention mechanism enhances the predictive performance of the Multi-input model when analyzing ECG signals from the SPH database, as shown in Table 8. The overall accuracy, average sensitivity, average PPV, and Macro-F1 score all saw improvements of approximately 0.49%, 0.46%, 0.46%, and 0.47%, respectively. Refer to Fig 10 for the confusion matrix of the Multi-input CNN model.

**Table 8:** Comparison of SE-multi-input CNN with Multi-input CNN.

Models	Overall Acc	Average Sen	Average PPV	Macro-F1
Multi-input CNN	95.35%	95.70%	95.24%	95.44%
SE-multi-input CNN	95.84%	96.16%	95.70%	95.91%

### 4.4. Comparison with state-of-the-art methods

To more accurately demonstrate the effectiveness of the algorithm, we compared the proposed multi-input CNN model with previous studies on arrhythmia classification, as shown in Table 9.

**Table 9:** Comparison with state-of-the-art arrhythmia classification.

Feature extraction method	# of classes	Performance reported(%)
CNN-LSTM +GA [45]	5	Acc = 98.00
		Sen = 99.70
		Spe = 98.90
		PPV = 95.80
		F1 = 89.7
CNN-LSTM + FFT [46]	5	Acc = 97.4
		Sen = 97.4
		PPV = 97.3
		F1 = 97.3
EasyEnsemble technique with global heartbeat [47]	4	Acc = 95.6
		Sen = 81.2
		PPV = 62.2
		F1 = 66.2
CNN-LSTM + FT [48]	5	Acc = 97.4
		Sen = 97.4
		PPV = 97.3
		F1 = 97.3
FFT + CNN-LSTM [49]	3	Acc = 99.2
		Sen = 99.2
		PPV = 99.2
		F1 = 99.2
CNN [50]	3	Acc = 98.52
		Sen = 98.52
		Spe = 99.26
		PPV = 98.55
		F1 = 98.52
STFT + 3-Channel CNN + GRU [51]	3	Acc = 99.8
		Sen = 99.8
		PPV = 99.8
		F1 = 99.8
STFT + Multi-input CNN + SE [current]	5	Acc = 99.13
		Sen = 92.26
		PPV = 97.07
		Macro-F1 = 94.46

Based on the comparative results presented in Table 9, we conclude that the proposed arrhythmia classification algorithm achieves competitive performance, demonstrating excellent accuracy and a high F1 score. Additionally, it can be observed that deep neural network models consistently achieve satisfactory results when applied to classification tasks [49–51].

## 5. Conclusion

This study proposes a novel arrhythmia classification framework that combines multi-scale ECG representations with a Squeeze-and-Excitation-enhanced CNN architecture. By jointly analyzing single-cycle and three-cycle STFT spectrograms, the model extracts both local and contextual features, while the integrated attention mechanism improves feature selectivity across channels. Extensive evaluations on the MIT-BIH and SPH arrhythmia datasets demonstrate that the proposed SE-multi-input CNN consistently outperforms state-of-the-art models in terms of accuracy, Macro-F1, and AUC.

### Clinical and practical implications

The ability to automatically and accurately classify arrhythmias using raw ECG signals has considerable implications for clinical decision support systems, especially in remote or resource-constrained environments. The proposed architecture, while lightweight compared to ensemble models, achieves high performance with reduced reliance on manual feature engineering. This makes it suitable for integration into portable ECG monitors or mobile health applications, enabling early detection and monitoring of cardiac conditions.

### Limitations and future work

Despite its effectiveness, the model has some limitations:

• Model complexity: The dual-branch architecture and STFT-based preprocessing increase computational overhead during inference, potentially limiting deployment on ultra-low-power devices.
• Generalizability: The model has been validated on two datasets with different structures (lead count, signal length), but further testing on additional real-world datasets (e.g., PhysioNet Challenge data) is needed to confirm robustness across broader clinical conditions.
• Fusion strategy: Although bicubic interpolation and summation offer a balance between performance and simplicity, more sophisticated fusion strategies (e.g., adaptive attention-based fusion or learnable concatenation layers) could further enhance feature integration and interpretability.

### Future research will explore

• End-to-end trainable pipelines that include R-peak detection, STFT generation, and feature fusion.
• Domain adaptation and transfer learning to handle patient variability.
• Explainable AI techniques to improve clinical trust in automated arrhythmia classifiers.
